# Impact of time between thrombolysis and endovascular thrombectomy on outcomes in patients with acute ischaemic stroke

**DOI:** 10.3389/fneur.2022.1018630

**Published:** 2022-11-02

**Authors:** Lora Wagner, Desiree Mohrbach, Martin Ebinger, Matthias Endres, Christian H. Nolte, Peter Harmel, Heinrich J. Audebert, Jessica L. Rohmann, Bob Siegerink

**Affiliations:** ^1^Center for Stroke Research Berlin, Charité—Universitätsmedizin Berlin, Berlin, Germany; ^2^Klinik für Neurologie mit Experimenteller Neurologie, Charité—Universitätsmedizin Berlin, Berlin, Germany; ^3^Klinik für Neurologie, Medical Park Berlin Humboldtmühle, Berlin, Germany; ^4^Berlin Institute of Health (BIH), Charité—Universitätsmedizin Berlin, Berlin, Germany; ^5^German Center for Neurodegenerative Diseases (DZNE), Partner Site Berlin, Berlin, Germany; ^6^German Centre for Cardiovascular Research (DZHK), Partner Site Berlin, Berlin, Germany; ^7^Institute of Public Health, Charité—Universitätsmedizin Berlin, Berlin, Germany; ^8^Department of Clinical Epidemiology, Leiden University Medical Center, Leiden University, Leiden, Netherlands

**Keywords:** ischaemic stroke, time-to-treatment, thrombolysis, thrombectomy, functional outcome, modified Rankin Scale, registry

## Abstract

**Background:**

Benefits of endovascular thrombectomy (ET) after intravenous thrombolysis (IVT) for patients with acute ischaemic stroke (AIS) have been demonstrated, but analyses of the relationship between IVT-ET time delay and functional outcomes among patients receiving both treatments are lacking.

**Methods:**

We used data from the “Berlin—Specific Acute Treatment in Ischaemic and haemorrhAgic stroke with Long-term outcome” (B–SPATIAL) registry. Between January 1st, 2016 and December 31st, 2019, we included patients who received both IVT and ET. The primary outcome was the 3-month ordinal modified Rankin scale (mRS) score. The IVT-ET time delay was analyzed in categories and continuously. We used adjusted ordinal logistic regression to estimate common odds ratios (cOR) and 95% confidence intervals (CI). Secondary analyses involved flexible modeling of IVT-ET delay and dichotomous outcomes.

**Results:**

Of 11,049 patients, 714 who received IVT followed by ET were included. Compared with having an IVT-ET window >120 min (reference), for an IVT-ET window < 30 min, we obtained adjusted cORs for mRS of 0.41 (95% CI: 0.22 to 0.78); and 0.52 (95% CI: 0.33 to 0.82) for 30 to 120 min. Secondary analyses also found protective effects of shorter time delays against “poor” functional outcomes at 3 months.

**Conclusions:**

In patients with AIS, shorter IVT-ET intervals were associated with better 3-month functional outcomes. While the time-to-IVT and time-to-ET include the time until medical attention is received, the IVT-ET time delays fall entirely within the domain of medical management and thus might be easier to optimize.

## Introduction

Acute ischaemic stroke (AIS) is one of the most common causes of morbidity and disability worldwide (1). There are two main acute treatment options for AIS, i.e., intravenous thrombolysis (IVT) and endovascular thrombectomy (ET) (2). In 2015, results from five randomized trials provided evidence for the superiority of ET, mostly in combination with IVT (“bridging thrombolysis”), compared to IVT alone (3–7). The benefits of IVT and ET combination therapy may be attributable to the ability of IVT to degrade remaining clot fragments, reduce ET procedure duration, and expedite recanalisation (8). Benefits of both recanalizing treatments, however, are known to diminish with increasing delay from symptom onset (or time last seen well) (9), hence, an earlier start of ET after IVT might result in more favorable outcomes for AIS patients.

Although “time–to–treatment” is a generally well-researched topic in stroke (10, 11), typically measured as the time of symptom onset to treatment initiation, the potential impact of the specific time delay between IVT and ET has not been well studied.

We aimed to estimate the effect of the time delay between IVT and ET on functional outcome as measured by the modified Rankin Scale (mRS) score 90 days after stroke among AIS patients who received both IVT and ET using prospectively-collected data from a large stroke registry in Berlin, Germany.

## Materials and methods

### Study design and setting

The Berlin—SPecific Acute Treatment in Ischemic or haemorrhAgic stroke with Long term follow–up (B–SPATIAL) registry (Clinicaltrials.gov identifier: NCT03027453) is a prospective, multicentre, observational registry of adult acute stroke and TIA patients presenting at one of 15 hospitals with stroke units in Berlin since January 1st, 2016. Patients aged 18 years or older of any sex with ICD-10 diagnoses of ischaemic stroke (I63), haemorrhagic stroke (I61), or Transient Ischaemic Attack (TIA) (G45.0–G45.3 and G45.5–G45.9) were eligible for inclusion in the registry. The registry includes data from patients transported to hospital by one of three Berlin mobile stroke units (MSUs) (12). Patients or their legal representatives were informed about the purpose and the procedures of the registry and had the opportunity to “opt–out” at multiple time points. Scientific evaluation of the B-SPATIAL registry was approved by the local ethics committee of the Charité–University Medicine Berlin (EA1/208/21).

The present study uses data collected through December 31st, 2019 by dedicated study nurses according to a standardized protocol, including hospital records and data from patient interviews or questionnaires. In cases of no response, information about patients' vital status was obtained via the city registry office 4 months after the index event (12).

In the present study, we restricted our sample to include only ischaemic stroke patients with symptom onset or time last seen well within 6 h of arrival at a participating hospital. We excluded patients with primary haemorrhagic stroke or TIA, as well as those with symptom remission before ambulance or hospital arrival, as they were not considered candidates for acute treatment (12). We included only patients who initiated both IVT and ET treatments in our analyses. Patients who received IVT while simultaneously undergoing ET (intra-arterial thrombolysis) were excluded.

### Patient characteristics

We obtained information about age, sex, blood pressure, blood glucose, and comorbidities, including atrial fibrillation, diabetes mellitus, and hypertension. In addition, we extracted clinical information including National Institutes of Health Stroke Scale (NIHSS) scores and vessel occlusion site (internal carotid artery, anterior cerebral artery, middle cerebral artery, and posterior cerebral artery).

### Exposure measures

The main exposure variable of interest, the elapsed time between IVT and ET (IVT-ET time delay), was computed as the difference between time of IVT initiation and time of ET initiation. In the analyses, we used both a primary clinical categorization (“short,” “medium,” “long”) of each time-to-treatment (time-to-IVT: < 60 min, 60–120 min, >120 min; time to ET: < 120 min, 120–280 min, >280 min; IVT-ET time delay: < 30 min, 30–120 min, >120 min), as well as a secondary exposure scale, in which we considered the IVT-ET time delay continuously, in 30-min incremental units.

### Outcome measures

Our primary outcome of interest was the functional outcome as defined by the modified Rankin Scale (mRS) score at 90 days after stroke. The mRS is a 7-point ordinal scale ranging from 0 (“no neurological symptoms”) to 6 (“death”) (13). In line with prior literature (3–7), we also present results using a dichotomous secondary outcome, modeling a “poor” (mRS: 3–6) vs. “favorable” (mRS: 0–2) functional outcome.

### Statistical analysis

We present medians and interquartile range limits (IQRL) for all continuous and ordinal variables, means and standard deviations for all normally distributed variables, and frequencies and percentages for categorical variables.

We used ordinal logistic regression (shift analysis) to obtain crude and adjusted common odds ratios (cOR) with corresponding 95% confidence intervals (95% CI) for the primary analysis. We further present results from crude and adjusted ordinal logistic regression models for the exposure variables time-to-IVT and time-to-ET.

The confounding adjustment strategy was determined *a priori* by selecting variables that are thought to be common causes of both the exposure and outcome or risk factors for the outcome. We included the following continuous variables in the adjusted models: age, NIHSS, blood pressure, blood glucose, and time-to-IVT, as well as the following categorical variables: sex, diagnosis of atrial fibrillation, diabetes mellitus, hypertension (or antihypertensive medication use), hospital size, time-to-IVT and vessel occlusion site. We created the variable “hospital size” to capture the relative sizes of the clinics as a proxy for structural factors such as geographic location, experience levels of the hospitals' physicians, treatment processes, and workflow. Of the 15 participating hospitals, five were included in each category: (1) treating < 4%, (2) Treating 4–10% or (3) treating >10% of all registry patients.

Missing values were assumed to be missing at random (MAR) and imputed using multiple imputation by chained equations (MICE) with 10 imputed datasets. The primary analyses were performed on the imputed datasets.

In the secondary analysis, we used logistic regression to estimate the effect of the IVT-ET time delay on the dichotomous 3-month functional outcome. To accommodate potential non-linear effects, IVT-ET time delay was modeled using splines (using *mksplines* in Stata) with knots set at every 30 min and using the 60–min knot as a reference. The 60-min reference was chosen based on the so-called “golden hour” of stroke, the time within which the initiation of reperfusion treatments are most effective for eligible acute ischemic stroke patients (14). This secondary analysis was performed in complete cases only (no imputation). We present a graphical representation of the binary odds ratio (OR) estimates for having a “poor” outcome (mRS>2) computed using multivariable logistic regression models adjusted for the aforementioned set of confounders.

Binary OR are commonly misinterpreted as being synonymous with relative risk, which can be especially problematic when the outcome is common (15). Since the prevalence of a “poor” outcome was approximately 50% in our study population, we opted to perform an additional modification to the aforementioned secondary analysis. We again used splines to model the IVT-ET time delay, this time obtaining adjusted relative risk (RR) estimates for the binary outcome using a modified Poisson regression modelling approach with robust standard errors (16). These results for having a “poor” functional outcome were also visualized with the 60-min knot as a reference.

All analyses were performed using STATA/IC 14 software (STATA Corp Ltd.).

## Results

### Study population

**Figure 1:**
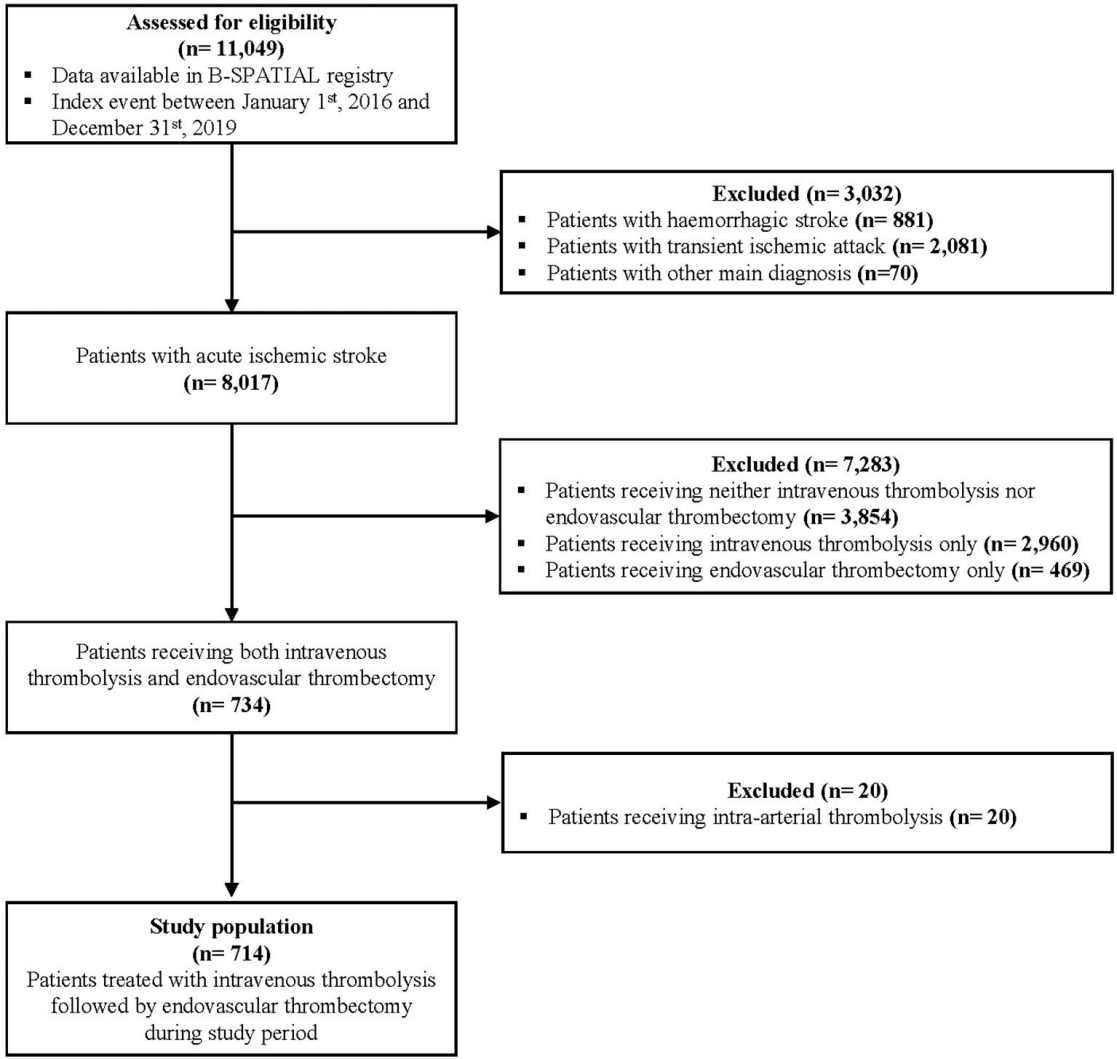
Flow diagram of study eligibility. B-SPATIAL, Berlin-Specific Acute Treatment in ischemic and haemorrhagic Stroke with Long-Term outcome.

Out of 11,049 patients meeting eligibility criteria for the B-SPATIAL registry between January 1st, 2016 and December 31st, 2019, a total of 714 patients treated with both IVT and ET were ultimately included in this study (Figure 1). Of these, 133 patients were transported by MSUs.

### Baseline characteristics

AIS patients receiving IVT and ET consisted primarily of elderly people suffering from moderate to severe strokes (Average age 72 years ±14 and median NIHSS score of 15 (IQRL 10–19). Fifty-one percent of those comprising the study population were female. A full summary of the baseline characteristics including times–to–treatment is displayed in Table 1.

**Table 1:** Baseline clinical and treatment characteristics of patients at hospital admission.

**Variable**	**Acute ischaemic stroke patients receiving intravenous thrombolysis then endovascular thrombectomy**
	**(*n* = 714)**
Patients transported with MSU, *n* (%)	133 (19)
Age, y, mean (SD)	72 (14)
median (IQRL)	75 (63–81)
Sex†, female, *n (%)*	364 (51)
Hospital size (based on percentage of registry patients treated)	
< 4%, *n (%)*	60 (8)
4–10%, *n (%)*	199 (28)
>10%, *n (%)*	455 (64)
Comorbidities	
Atrial fibrillation†, *n (%)*	271 (38)
Diabetes mellitus†, *n (%)*	161 (23)
Hypertension†, *n (%)*	560 (78)
NIHSS†, median (IQRL)	15 (10–19)
Systolic blood pressure ‡, mmHg, mean (SD)	156 (30)
Diastolic blood pressure ‡, mmHg, mean (SD)	85 (17)
Blood glucose ‡, mg/dl, mean (SD)	135 (42)
Vessel occlusion site	
Internal carotid artery, *n (%)*	76 (11)
Anterior cerebral artery, *n (%)*	18 (3)
Middle cerebral artery, *n (%)*	454 (63)
Posterior cerebral artery, *n (%)*	23 (3)
Other or no information available, *n* (%)	143 (20)
Time from symptom onset to IVT, mins, mean (SD),	112 (64)
median (IQRL)	90 (68–135)
Time from symptom onset to ET, mins, mean (SD),	194 (131)
median (IQRL)	169 (130–224)
Time between IVT and ET, mins, mean (SD),	82 (116)
median (IQRL)	66 (44–92)

SD, standard deviation; IQRL, interquartile range limits; NIHSS, National Institutes of Health Stroke Scale; IVT, intravenous tissue–type plasminogen activator; ET, Endovascular Thrombectomy; MSU, Mobile Stroke Unit; †variable had < 10% missing values; ‡variable had >10% missing values.

43 (6%) patients had already experienced prior ischemic stroke or TIA according to their medical documentation. 67 (9%) patients developed a symptomatic secondary intracerebral hemorrhage, and 54 (8%) patients died in–hospital. The mRS 90 days after the index event was available for 573 (80%) patients, with a median value of 3 (IQRL 1–5).

We also provide baseline characteristics stratified by IVT-ET time delay groups in Supplementary Table S1.

### Impact of IVT-ET time delay on functional outcome

In the primary analysis, after confounding adjustment, we found that having a “short” or “medium” time delay between IVT and ET was associated with a favorable shift in the distribution of mRS scores (shift to lower scores) 90 days after AIS compared with having a “long” time delay (Table 2). Compared to having an IVT-ET time delay of >120 min (reference), for an IVT-ET time delay of fewer than 30 min, we obtained a beneficial adjusted cOR of 0.41 (95% CI 0.22 to 0.78), and for IVT-ET time delays between 30 to 120 min, an adjusted cOR of 0.52 (95% CI 0.33 to 0.82).

**Table 2:** Ordinal logistic regression results, effect estimates for IVT-ET time delay on mRS score 90 days after index acute ischaemic stroke event.

	**mRS at 90 days**	
**Time delay between intravenous thrombolysis and endovascular thrombectomy**	**Unadjusted cOR (95% CI)**	**Adjusted cOR†(95% CI)**
Primary exposure categorization		
< 30 mins (*n =* 71)	0.64 (0.35 to 1.17)	0.41 (0.22 to 0.78)
30–120 mins (*n =* 551)	0.71 (0.46 to 1.10)	0.52 (0.33 to 0.82)
>120 mins (*n =* 92)	1 (reference)	1 (reference)
Exposure as a continuous variable		
per 30-min reduction in IVT-ET time delay	0.97 (0.92 to 1.02)	0.94 (0.88 to 1.00)

cOR, common Odds ratio obtained from the ordinal logistic regression models for each exposure category; CI, Confidence interval; IVT, Intravenous Thrombolysis; ET, Endovascular Thrombectomy; mRS, modified Rankin Scale.

†Adjusted for, age, sex, NIHSS, blood pressure, blood glucose, atrial fibrillation, diabetes mellitus, hypertension, hospital size, vessel occlusion site, and time-to-IVT.

Treating the exposure as a continuous variable in the primary analysis, each 30-min reduction in time delay was found to be associated with a favorable shift in the distribution of mRS based on the point estimate, though this result was not statistically significant (adjusted cOR of 0.94, 95% CI 0.88 to 1.00).

The corresponding results for the time-to-IVT and time-to-ET exposures are described and presented in the Supplementary Results and Supplementary Table S2.

**Figure 2:**
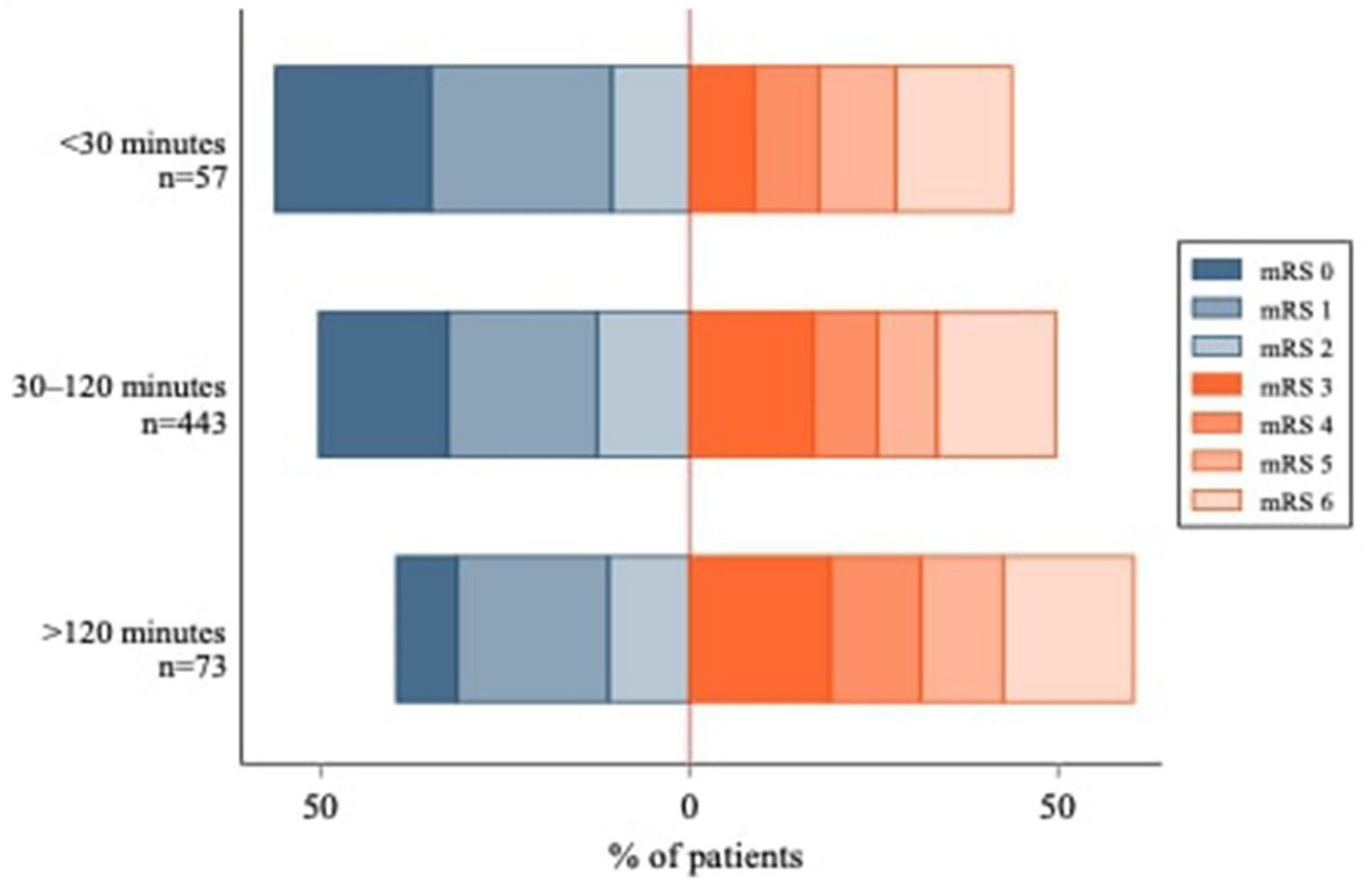
Modified Rankin Scale Score Distribution at 90 days by IVT-ET time delay group. The IVT-ET time delay corresponds to the elapsed time between intravenous thrombolysis (IVT) and endovascular thrombectomy (ET). 90-day mRS scores were not available for 141 patients.

**Figure 3:**
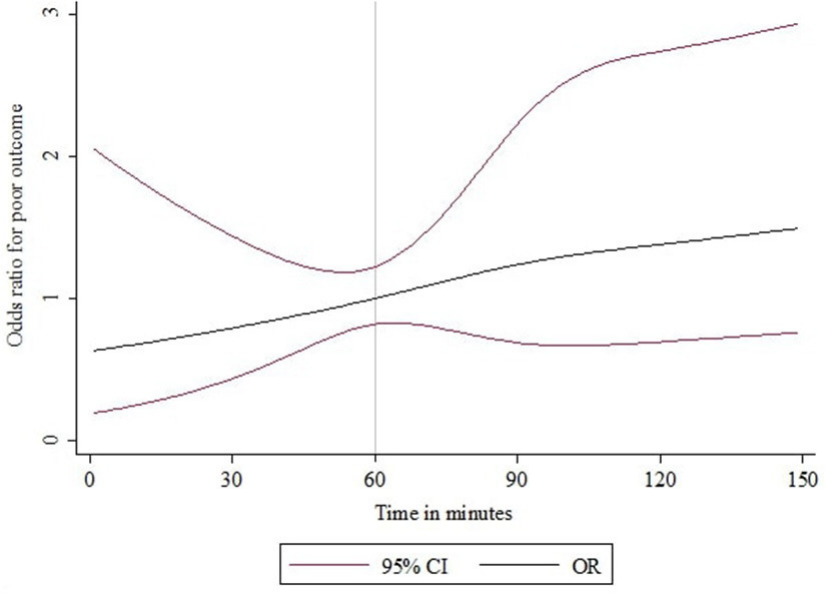
IVT-ET time delay and binary OR for “poor” functional outcome (mRS > 2) at 90 days after acute ischaemic stroke. The time delay between IVT and ET was modeled as a continuous exposure variable using splines and the odds ratio estimates are for a “poor” functional outcome (mRS 3–6). An IVT-ET time delay of 60 elapsed minutes was used as the reference.

Figure 2 shows the unadjusted mRS distribution across the three primary IVT-ET time delay groups. As depicted, the shorter the IVT-ET time delay, the more favorable the shift toward lower 90-day mRS scores. Similarly, Figure 3 shows a higher adjusted binary OR for “poor” 90-day functional outcome with increasing time delay between IVT and ET. Supplementary Figure S1 shows a modified version of Figure 3 using risk ratios instead of ORs.

## Discussion

Our findings provide a detailed analysis of the relationship between the elapsed delay in time between IVT and ET and functional outcomes in ischaemic stroke patients who received both treatments. Our results indicate that shorter IVT-ET time delays are associated with better functional outcomes 3 months after ischaemic stroke, corresponding to a favorable shift in the distribution of mRS scores. These effect estimates are similar to the protective effects for reducing time-to-IVT or time-to-ET treatment in both direction and magnitude. Consistent with our findings, prior research has consistently demonstrated detrimental effects of longer time-to-IVT and time-to-ET in different settings (10, 11, 17).

Rapid initiation of ET following IVT appears to have a considerable protective benefit, whereas longer delays between treatments appear to be detrimental in terms of longer-term functional outcomes. Our findings corroborate those of the French Endovascular Treatment in Ischemic Stroke (ETIS) registry (18). Zhu et al. analyzed 1,986 AIS patients in six comprehensive stroke centers, and found that having a longer IVT-ET time delay was associated with a worse functional outcome at 90 days (adjusted OR for the favorable outcome (mRS 0–2) per 30-min increase = 0.91, 95% CI 0.86 to 0.96) (18). Findings from Evans et al. (19) indicated a significant benefit of ET within 90 minutes after IVT (20) in a *post-hoc* subgroup analysis of the IMS III trial.

Recent studies mainly conducted in Europe, North America and Australia suggest that IVT before ET has a beneficial effect, even in patients treated with ET (21). IVT followed by ET was associated with higher reperfusion rates without a significantly higher rate of symptomatic intracranial hemorrhage compared to ET alone. We unfortunately do not have data on the experience level of individual ET operators at the hospitals participating in the B-SPATIAL registry. However, the number of ET treatments performed in the participating hospitals (also counting ETs without prior IVT) ranged from 1 to 238 during the included time period. In two hospitals, the thrombectomy service was first introduced at the end of the inclusion period, explaining the low numbers of ET treatments seen in those two hospitals. Therefore, we assume that most interventionalists were rather experienced.

When interpreting our findings, readers should consider our study's strengths and limitations. At the time of analysis, the B–SPATIAL registry comprised more than 10,000 stroke patients, yet < 10% ultimately received both IVT and ET, meeting our inclusion criteria (714 AIS patients). Despite this being a sufficient sample size for our chosen analyses, we did not have enough power to assess potential effect modification.

International and national efforts such as Safe Implementation of Thrombolysis (SITS), International Stroke Thrombolysis Register, the German Stroke Registry, or The China National Stroke Registry (CNSR) (22–24) may have reached higher numbers than our Berlin-based registry, but differences in data collection between hospitals can affect the quality and quantity of their collected data and follow–up of functional outcome is often incomplete. Our analyses benefitted from the comprehensive and systematic data collection, which was actively promoted in the B-SPATIAL registry through the use of standardized protocols and trained, dedicated study nurses in all hospitals with a stroke unit in Berlin, Germany.

Of note, our study population did not include patients who had recanalisation of their large vessel occlusion through IVT alone. This selection may have led to an overrepresentation of patients with worse outcomes, since rapidly resolving cases did not meet our inclusion criteria.

Finally, we further acknowledge that the routinely collected variables included as covariates may not have fully captured potential causes of IVT-ET time delays that are also causes of functional outcomes. A potential source of residual confounding we considered important is hospital organisation. This includes the hospital's geographic location, angiography facility, diagnostic and therapeutic workflows, and other structural elements. However, since these factors are difficult to quantify, they could not be individually captured in the registry. For this reason, we opted to adjust for hospital size as a proxy variable. Indeed, the exact causes of IVT-ET time delays likely differ per setting. Once identified and implemented, targeted improvement measures are likely to reduce delays and subsequently impact patient outcomes.

Our findings indicate a meaningful relationship between the time delay between IVT and ET and the functional outcomes of ischaemic stroke patients three months after stroke. After accounting for confounding including time-to-IVT, our effect estimates are similar in magnitude to published effect estimates from the literature for time-to-IVT or time-to-ET. While the time-to-IVT and time-to-ET include the time until medical attention is received (e.g., response following emergency call), and are therefore difficult to modify, the elapsed time between IVT and ET falls entirely within the domain of medical management and thus might be easier to optimize.

## Data availability statement

The data analyzed in this study was obtained from the Berlin - Specific Acute Treatment in Ischemic or haemorrhAgic stroke with Long term follow-up (B-SPATIAL) registry, the following licenses/restrictions apply: The datasets presented in this article are not readily available because of data protection regulations. Data can be made available in a de-identified manner to researchers upon reasonable request (to the extent allowed by the registry's data protection agreement). Requests to access these datasets should be directed to Jessica L. Rohmann, jessica.rohmann@charite.de.

## Ethics statement

The scientific evaluation of B-SPATIAL registry data was approved by the Ethics Committee of Charité-Universitätsmedizin Berlin (EA1/208/21). The B-SPATIAL registry used an opt-out mechanism for patient inclusion. Two months after their index event, patients were informed in writing about the inclusion of their record in the B-SPATIAL registry and had multiple opportunities to opt out.

## Author contributions

LW, HA, and BS conceptualized the study. MEb, MEn, CN, PH, and HA were involved in data acquisition. The statistical analyses were planned and conducted by LW and BS, also in consultation with DM and JR. LW, HA, JR, and BS interpreted the results and drafted the manuscript. LW created all figures and tables. HA provided funding for the project and as well as project supervision together with BS and JR. All authors critically revised the final version of the manuscript.

## Funding

The B-SPATIAL registry was funded by the German Federal Ministry of Education and Research and the German Research Foundation (DFG) granted to the Center for Stroke Research Berlin. MEn further reports funding from the DFG under Germany's Excellence Strategy - EXC-2049 - 390688087. The funders had no role in study design, analysis of the data, interpretation of the results, or drafting of the text.

## Conflict of interest

Author JR reports receiving a grant from Novartis Pharma for a self-initiated research project on migraine remission unrelated to this work. Author MEn reports receiving grants from Bayer and fees paid to the Charité-Universitätsmedizin Berlin from AstraZeneca, Bayer, BMS, Pfizer, Daiichi Sankyo, Amgen, GSK, Sanofi, Covidien, and Novartis, outside of this work. Author CN received research grants from German Ministry of Research and Education, German Center for Neurodegenerative Diseases, German Center for Cardiovascular Research, and speaker and/or consultation fees from Boehringer Ingelheim, Bristol-Myers Squibb, Pfizer Pharma, Daiichi Sankyo, Alexion, Abbott, and Bayer. Author HA received research grants from German Ministry of Research and Education and the German Research Foundation for the B-SPATIAL registry and the B_PROUD study and reports personal fees from Bayer Vital, Boehringer Ingelheim, Bristol- Myers Squibb, Novo Nordisk, Pfizer, and from Sanofi outside the submitted work. The remaining authors declare that the research was conducted in the absence of any commercial or financial relationships that could be construed as a potential conflict of interest.

## Publisher's note

All claims expressed in this article are solely those of the authors and do not necessarily represent those of their affiliated organizations, or those of the publisher, the editors and the reviewers. Any product that may be evaluated in this article, or claim that may be made by its manufacturer, is not guaranteed or endorsed by the publisher.

## References

[1] GBD 2019 Stroke Collaborators. Global, regional, and national burden of stroke and its risk factors, 1990-2019: a systematic analysis for the Global Burden of Disease Study 2019. Lancet Neurol. (2021) 20:795–820. 10.1016/S1474-4422(21)00252-0 34487721 PMC8443449

[2] Ciccone A Valvassori L Nichelatti M Sgoifo A Ponzio M Sterzi R Boccardi E SYNTHESIS Expansion Investigators. Endovascular treatment for acute ischemic stroke. N Engl J Med. (2013) 368:904–13. 10.1056/NEJMoa1213701 23387822 PMC3708480

[3] Goyal M Demchuk AM Menon BK Eesa M Rempel JL Thornton J. ESCAPE Trial Investigators. Randomized assessment of rapid endovascular treatment of ischemic stroke. N Engl J Med. (2015) 372:1019–30. 10.1056/NEJMoa1414905 25671798

[4] Berkhemer OA Fransen PS Beumer D van den Berg LA Lingsma HF Yoo AJ. MR CLEAN Investigators. A randomized trial of intraarterial treatment for acute ischemic stroke. N Engl J Med. (2015) 372:11–20. 10.1056/NEJMoa1411587 25517348

[5] Campbell BC Mitchell PJ Kleinig TJ Dewey HM Churilov L. EXTEND-IA Investigators. Endovascular therapy for ischemic stroke with perfusion-imaging selection. N Engl J Med. (2015) 372:1009–18. 10.1056/NEJMoa1414792 25671797

[6] Jovin TG Chamorro A Cobo E de Miquel MA Molina CA Rovira A. REVASCAT Trial Investigators. Thrombectomy within 8 hours after symptom onset in ischemic stroke. N Engl J Med. (2015) 372:2296–306. 10.1056/NEJMoa1503780 25882510

[7] Saver JL Goyal M Bonafe A Diener HC Levy EI Pereira VM. SWIFT PRIME Investigators. Stent-retriever thrombectomy after intravenous t-PA vs t-PA alone in stroke. N Engl J Med. (2015) 372:2285–95. 10.1056/NEJMoa1415061 25882376

[8] Ferrigno M Bricout N Leys D Estrade L Cordonnier C Personnic T. Intravenous recombinant tissue-type plasminogen activator: influence on outcome in anterior circulation ischemic stroke treated by mechanical thrombectomy. Stroke. (2018) 49:1377–85. 10.1161/STROKEAHA.118.020490 29748424

[9] Lees KR Bluhmki E von Kummer R Brott TG Toni D Grotta JC. ECASS, ATLANTIS, NINDS and EPITHET rt-PA Study Group. Time to treatment with intravenous alteplase and outcome in stroke: an updated pooled analysis of ECASS, ATLANTIS, NINDS, and EPITHET trials. Lancet. (2010) 375:1695–703. 10.1016/S0140-6736(10)60491-6 20472172

[10] Saver JL Goyal M van der Lugt A Menon BK Majoie CB Dippel DW. HERMES Collaborators. Time to treatment with endovascular thrombectomy and outcomes from ischemic stroke: a meta-analysis. JAMA. (2016) 316:1279–88. 10.1001/jama.2016.13647 27673305

[11] Emberson J Lees KR Lyden P Blackwell L Albers G Bluhmki E. Stroke Thrombolysis Trialists' Collaborative Group. Effect of treatment delay, age, and stroke severity on the effects of intravenous thrombolysis with alteplase for acute ischaemic stroke: a meta-analysis of individual patient data from randomised trials. Lancet. (2014) 384:1929–35. 10.1016/S0140-6736(14)60584-5 25106063 PMC4441266

[12] Kunz A Ebinger M Geisler F Rozanski M Waldschmidt C Weber JE. Functional outcomes of pre-hospital thrombolysis in a mobile stroke treatment unit compared with conventional care: an observational registry study. Lancet Neurol. (2016) 15:1035–43. 10.1016/S1474-4422(16)30129-6 27430529

[13] Kasner SE. Clinical interpretation and use of stroke scales. Lancet Neurol. (2006) 5:603–12. 10.1016/S1474-4422(06)70495-1 16781990

[14] Ebinger M Kunz A Wendt M Rozanski M Winter B Waldschmidt C. Effects of golden hour thrombolysis: a Prehospital Acute Neurological Treatment and Optimization of Medical Care in Stroke (PHANTOM-S) substudy. JAMA Neurol. (2015) 72:25–30. 10.1001/jamaneurol.2014.3188 25402214

[15] Knol MJ Cessie SL Algra A Vandenbroucke JP Groenwold RHH. Overestimation of risk ratios by odds ratios in trials and cohort studies: alternatives to logistic regression. CMAJ. (2012) 184:895–9. 10.1503/cmaj.101715 22158397 PMC3348192

[16] Zou G A. modified poisson regression approach to prospective studies with binary data. Am J Epidemiol. (2004) 159:702–6. 10.1093/aje/kwh090 15033648

[17] Fransen PS Berkhemer OA Lingsma HF Beumer D van den Berg LA Yoo AJ. Multicenter Randomized Clinical Trial of Endovascular Treatment of Acute Ischemic Stroke in the Netherlands Investigators. Time to reperfusion and treatment effect for acute ischemic stroke: a randomized clinical trial. JAMA Neurol. (2016) 73:190–6. 10.1001/jamaneurol.2015.3886 26716735

[18] Zhu F Gauberti M Marnat G Bourcier R Kyheng M Labreuche J. ETIS Registry Investigators. Time from IV thrombolysis to thrombectomy and outcome in acute ischemic stroke. Ann Neurol. (2021) 89:511–9. 10.1002/ana.25978 33274475

[19] Evans MRB White P Cowley P Werring DJ. Revolution in acute ischaemic stroke care: a practical guide to mechanical thrombectomy. Pract Neurol. (2017) 17:252–65. 10.1136/practneurol-2017-001685 28647705 PMC5537551

[20] Demchuk AM Goyal M Yeatts SD Carrozzella J Foster LD Qazi E. IMS III Investigators. Recanalization and clinical outcome of occlusion sites at baseline CT angiography in the Interventional Management of Stroke III trial. Radiology. (2014) 273:202–10. 10.1148/radiol.14132649 24895878 PMC4174723

[21] Turc G Tsivgoulis G Audebert HJ Boogaarts H Bhogal P De Marchis GM. European Stroke Organisation - European Society for Minimally Invasive Neurological Therapy expedited recommendation on indication for intravenous thrombolysis before mechanical thrombectomy in patients with acute ischaemic stroke and anterior circulation large vessel occlusion. Eur Stroke J. (2022) 7:I–XXVI. 10.1177/23969873221076968 35300256 PMC8921785

[22] Keselman B Cooray C Vanhooren G Bassi P Consoli D Nichelli P. Intravenous thrombolysis in stroke mimics: results from the SITS international stroke thrombolysis register. Eur J Neurol. (2019) 26:1091–7. 10.1111/ene.13944 30793434

[23] Alegiani AC Dorn F Herzberg M Wollenweber FA Kellert L Siebert E. Systematic evaluation of stroke thrombectomy in clinical practice: the German STROKE Registry endovascular treatment. Int J Stroke. (2019) 14:372–80. 10.1177/1747493018816194 30346260

[24] Wang Y Cui L Ji X Dong Q Zeng J Wang Y. China National Stroke Registry Investigators. The China National Stroke Registry for patients with acute cerebrovascular events: design, rationale, and baseline patient characteristics. Int J Stroke. (2011) 6:355–61. 10.1111/j.1747-4949.2011.00584.x 21609414

