# Supplementary material for: Impact of time between thrombolysis and endovascular thrombectomy on outcomes in patients with acute ischaemic stroke
Source: Front Neurol. 2022 Nov 2;13:1018630. doi: 10.3389/fneur.2022.1018630 (PMC9667508; doi:10.3389/fneur.2022.1018630)
Supplement: Supplementary file 1 [file Data_Sheet_1.pdf]

Supplementary Material

1 Supplemental Results: Time-to-IVT, time-to-ET and functional outcome among AIS patients receiving both treatments

Our results indicate that reductions in time to treatment for both IVT and ET are associated with favorable shifts in the distribution of mRS functional outcomes. For each 30 minutes of reduction in times to treatment, the adjusted common OR for mRS at 90 days was 0.93 (95% CI 0.86 to 1.00) for IVT, and 0.94 (95% CI 0.89 to 0.99) for ET. The effect estimates for different time categories are more pronounced: the adjusted cOR for early IVT (<60 minutes from symptom onset or time last seen well) was 0.54 (95% CI 0.34 to 0.88) whereas the adjusted cOR for IVT within 60–120 minutes was 0.83 (95% CI 0.60 to 1.15), both compared to the late category of >120 minutes. The adjusted cOR for time-to-ET was 0.37 (95% CI 0.20 to 0.67) for <120 minutes from symptom onset or time last seen well and 0.60 (95% CI 0.38 to 0.96) for 120–280 minutes, each compared to the late reference category (>280 minutes).

2 Supplementary Tables

Supplemental Table 1. Baseline clinical and treatment characteristics of patients at hospital admission according to IVT-ET time delay

	IVT-ET time delay		
Variable	<30 minutes	30-120 minutes	>120 minutes
Numbers of patients	71	551	92
Patients transported with MSU, n (%)	4 (6)	103 (19)	26 (28)
Age, y, mean (SD)<br>median (IQRL)	70 (15)<br>70 (58–81)	72 (14)<br>75 (63–81)	72 (13)<br>75 (65–82)
Sex†, female, n (%)	35 (49)	287 (52)	42 (46)
Hospital size (based on percentage of registry patients treated)<br><4%, n (%)<br>4-10%, n (%)<br>>10%, n (%)	 5 (7)<br>16 (23)<br>50(70)	 48 (9)<br>154 (28)<br>349 (63)	 7 (8)<br>29 (32)<br>56 (61)
Comorbidities<br>Atrial fibrillation†, n (%)<br>Diabetes mellitus†, n (%)	 24 (34)<br>18 (25)	 206 (38)<br>123 (22)	 41 (45)<br>20 (22)

Hypertension†, n (%)	56 (79)	426 (77)	78 (85)
NIHSS†, median (IQRL)	16 (13 - 20)	15 (10 - 19)	13 (7 - 19)
Systolic blood pressure ‡, mmHg, mean (SD)	156 (30)	155 (29)	156 (26)
Diastolic blood pressure ‡, mmHg, mean (SD)	85 (15)	85 (17)	86 (16)
Blood glucose ‡, mg/dl, mean (SD)	136 (41)	135 (42)	134 (41)
Vessel occlusion site			
Internal carotid artery, n (%)	5 (7)	64 (12)	7 (8)
Anterior cerebral artery, n (%)	4 (6)	11 (2)	3 (3)
Middle cerebral artery, n (%)	35 (49)	359 (65)	60 (65)
Posterior cerebral artery, n (%)	5 (7)	15 (3)	3 (3)
Other or no information given, n (%)	22 (31)	102 (18)	19 (21)
Time from symptom onset to IVT, minutes, median (IQRL)	126<br>108 (74 - 170)	111<br>89 (67 - 135)	105<br>91 (70 - 129)
Time from symptom onset to ET, minutes, median (IQRL)	145<br>125 (95 - 185)	178<br>163 (130 - 205)	330<br>257 (219 - 314)
Time between IVT and ET, minutes, median (IQRL)	19<br>20 (11 - 26)	67<br>65 (49 - 83)	225<br>144 (133 - 179)

Supplemental Table 2. Ordinal logistic regression results: effect estimates for symptom onset to Intravenous Thrombolysis and symptom onset to Endovascular Thrombectomy on mRS score 90 days after index acute ischaemic stroke event

	mRS at 90 days	
	unadjusted cOR (95% CI)	adjusted cOR* (95% CI)
Time between symptom onset and Intravenous Thrombolysis		
Primary exposure categorization		
<60 min (n=104)	0.46 (0.30–0.72)	0.54 (0.34–0.88)
60–120 min (n=391)	0.82 (0.60–1.13)	0.83 (0.60–1.15)
>120 min (n=219)	1 –reference-	1 –reference-

Exposure as a continuous variable		
per 30-minute reduction in time to treatment	0.92 (0.86–0.98)	0.93 (0.86–1.00)
Time between symptom onset and Endovascular Thrombectomy		
Primary exposure categorization		
<120 min (n=112)	0.37 (0.21–0.65)	0.37 (0.20–0.67)
120–280 min (n=520)	0.72 (0.46–1.11)	0.60 (0.38–0.96)
>280 min (n=82)	1 –reference-	1 –reference-
Exposure as a continuous variable		
per 30-minute reduction in time to treatment	0.95 (0.91–1.00)	0.94 (0.89–0.99)

cOR: common Odds ratio obtained from the ordinal logistic regression models for each exposure category; CI: Confidence interval; mRS: modified Rankin Scale.

*adjusted for: age, sex, NIHSS, blood pressure, blood glucose, atrial fibrillation, diabetes mellitus, hypertension, hospital size, vessel occlusion site, and time-to-IVT. See Methods for detailed variable descriptions and categorizations.

2.1 Supplementary Figures

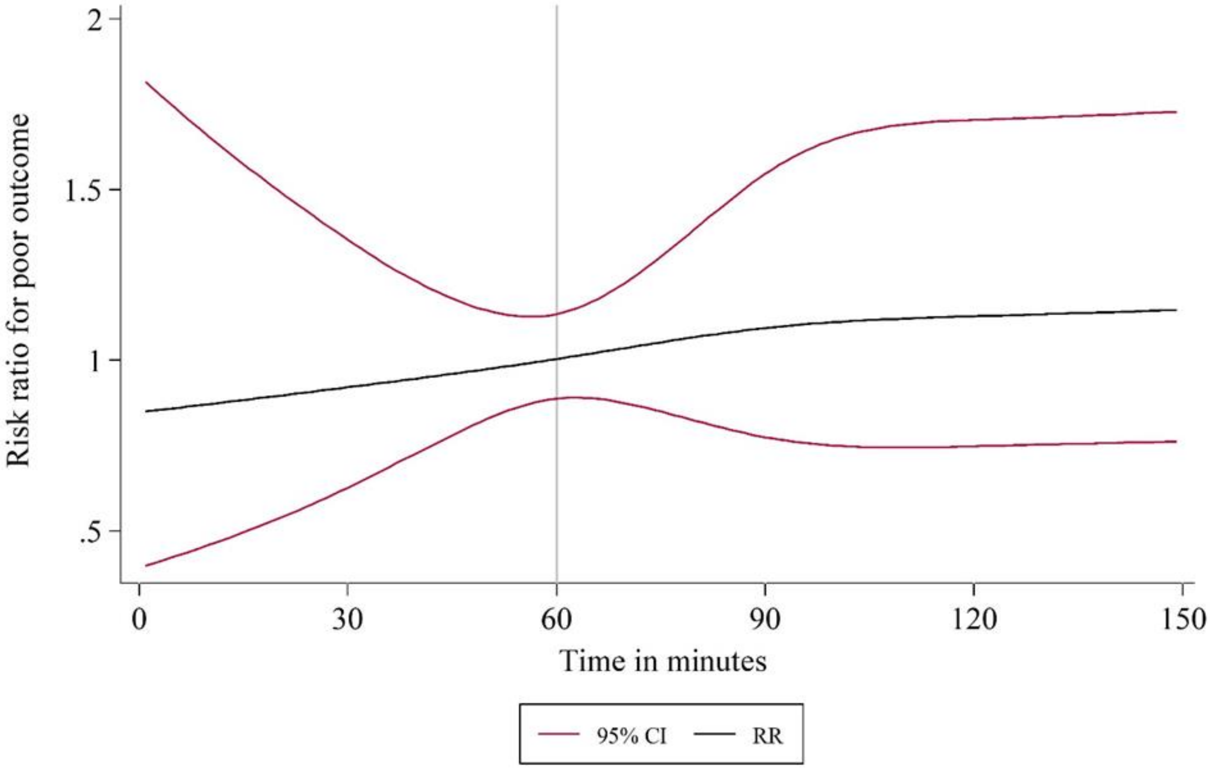

Supplemental Figure 1. IVT-ET time delay and binary risk ratio for "poor" functional outcome (mRS > 2) at 90 days after acute ischaemic stroke.

The time between IVT and ET was modelled as a continuous exposure variable using splines. An IVT-ET time delay of 60 elapsed minutes is the reference. The displayed risk ratio (RR) estimates for a "poor" functional outcome (mRS > 2) were obtained from a generalized linear model (modified Poisson approach) adjusted for age, sex, NIHSS, blood pressure, blood glucose, atrial fibrillation, diabetes mellitus, hypertension, hospital size, vessel occlusion site, and the time-to-IVT in a complete case analysis (n=562).
